# Supplementary material for: In-vitro comparative thermo-chemical aging and penetration analyses of bioactive glass-based dental resin infiltrates
Source: PeerJ. 2025 Jan 28;13:e18831. doi: 10.7717/peerj.18831 (PMC11784535; doi:10.7717/peerj.18831)
Supplement: Supplemental Information 1 [file peerj-13-18831-s001.pdf]

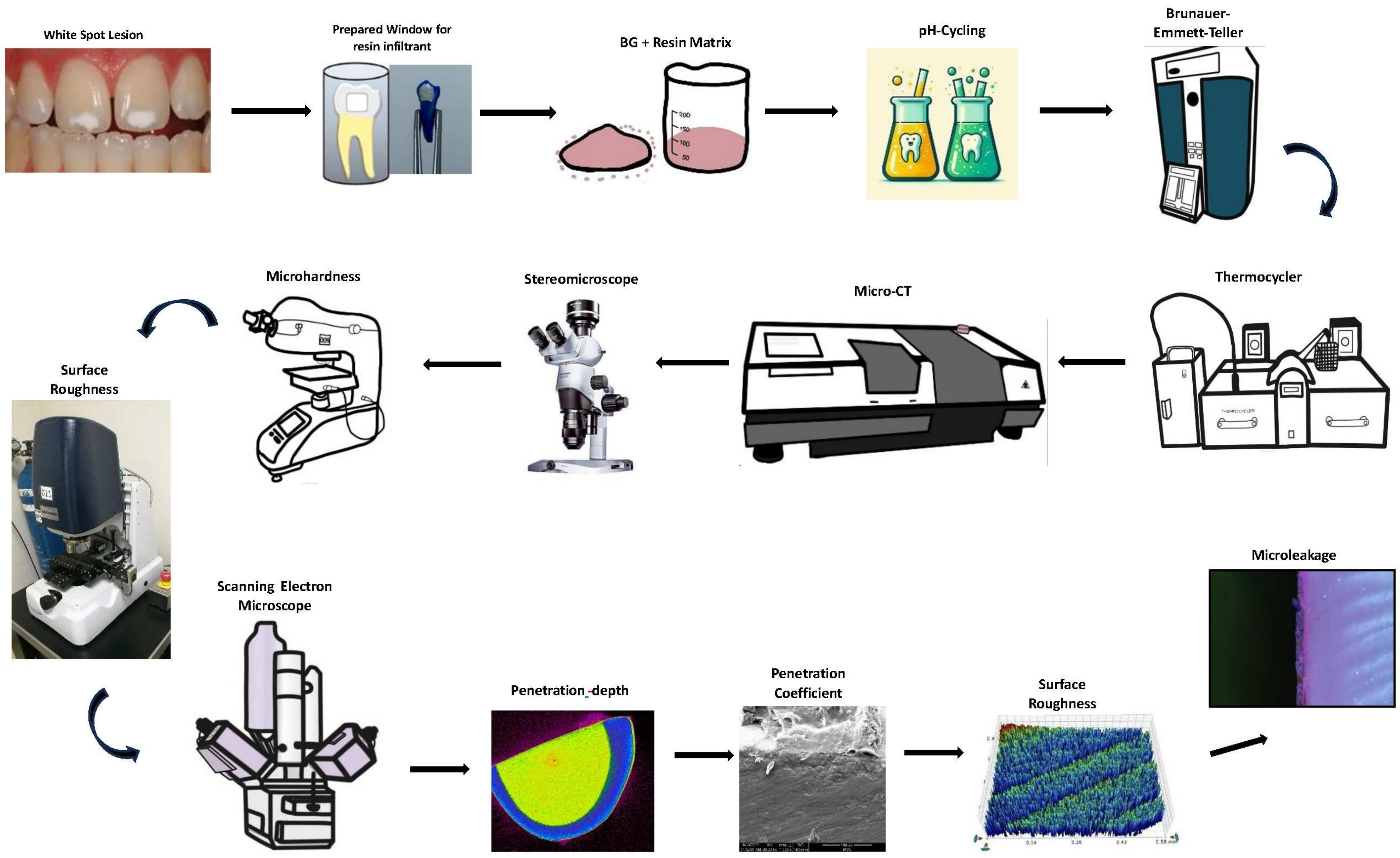
White Spot Lesion
Prepared Window for resin infiltrant
BG + Resin Matrix
pH-Cycling
Brunauer-Emmett-Teller
Thermocycler
Micro-CT
Stereomicroscope
Microhardness
Surface Roughness
Scanning Electron Microscope
Penetration -depth
Penetration Coefficient
Surface Roughness
Microleakage
