# Supplementary material for: In-vitro comparative thermo-chemical aging and penetration analyses of bioactive glass-based dental resin infiltrates
Source: PeerJ. 2025 Jan 28;13:e18831. doi: 10.7717/peerj.18831 (PMC11784535; doi:10.7717/peerj.18831)
Supplement: Supplemental Information 2 — Most of the characterizations were performed along with positive control (ICON resin infiltrant) however, in some of them, ICON was not utilized. [file peerj-13-18831-s002.docx]

**ICON RESIN INFILTRANT**

**Technique**

Keep the tooth structure dry and clear of any debris. The commercially available resin infiltrant (ICON), which served as the study's positive control, was applied according to the manufacturer's specified standard methodology and application guidelines. The enamel pores were first etched for two minutes (120 A window of 4mm × 4mm was created on the enamel surfaces and the remaining area was covered with nail polish. To create the artificial white spot lesion, the samples were submerged in a demineralization solution for 96 hours at 37°C. The demineralization solution was prepared by 0.05 mM acetic acid solution, 2.2 mM CaCl2, and 2.2 mM Na3PO4 and a few drops of KOH was adjusted, and the pH was finally corrected to 4.4.

*Table S1*: Composition of Resin infiltrant groups along with filler

| **Groups** | **Composition** | **Filler type** | **Filler Percentage** |
| --- | --- | --- | --- |
| ICON | TEGDMA-Based + Additives | None | None |

**Microhardness – Surface Roughness (Chemical Aging /Thermocycling)**

*Table S2*: Analytic Microhardness and Surface Roughness values of All the Resin Infiltrated teeth samples with Mean and Standard Deviation after immersing into the Chemical ageing solution and Thermocycling process. The last two columns show the Microleakage and Penetration depth values for ICON.

| Groups | **Vickers Microhardness** | **Surface Roughness** | **Microleakage (µm)** | **Penetration Depth (µm)** |
| --- | --- | --- | --- | --- |
| Pre-Chemical ageing | 137.35±0.97 | 665.56± 19.22 | 4.25±0.14 | 4.81±0.19 |
| Post Chemical ageing | 111.9±4.33 | 969.71± 4.43 |  |  |
| Pre-Thermocycling | 157.43± 17.06 | 0.73±0.04 |  |  |
| Post-Thermocycling | 147.63± 16.0 | 0.96±0.004 |  |  |
|  | <0.001 | <0.001 |  |  |

**SEM - Thermocycling**


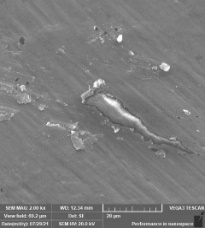

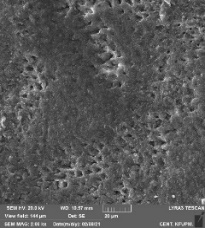


**A**

**B**

**(b)**

**(a)**

*Fig. S1*: Resin Infiltrant material teeth surface with the process of thermocycling. Fig. S1(A) and S1(B) ICON resin, baseline scans before thermocycling and after thermocycling.

**SEM - Chemical Ageing**


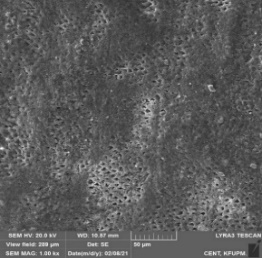


**A**


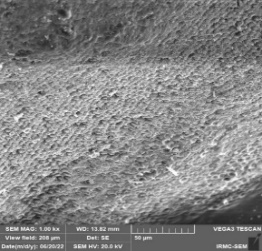


**B**

*Fig. S2*: Resin Infiltrant material on teeth into the Chemical ageing solution. Fig. S2(A) & Fig. S2(B), ICON scans before and after immersion into the chemical solution.

**Microleakage**


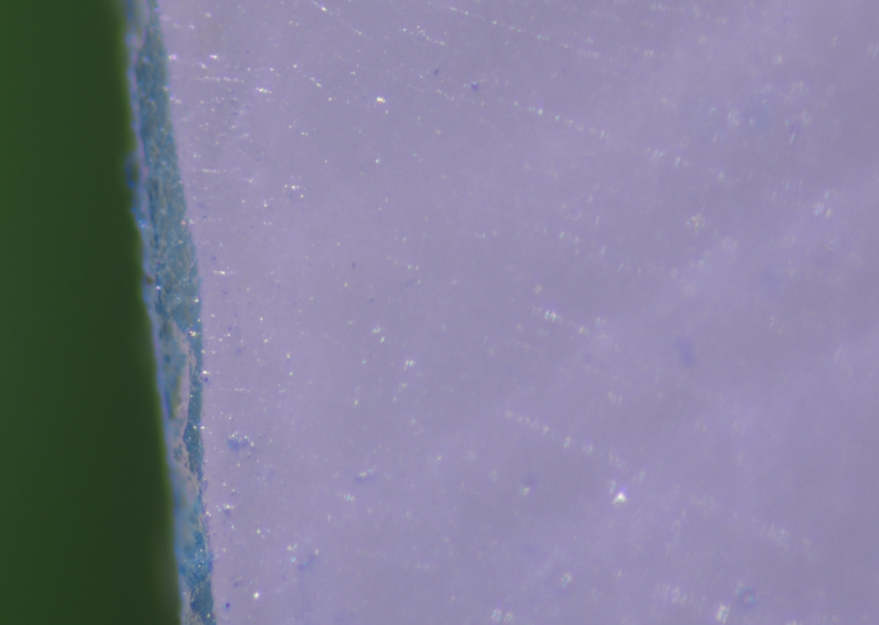


*Fig. S3*: Microleakage analysis of teeth surfaces with ICON resin infiltrant. Stereomicroscope images after immersing into the Methylene Blue dye.

**Penetration Depth / Micro-CT Analysis**


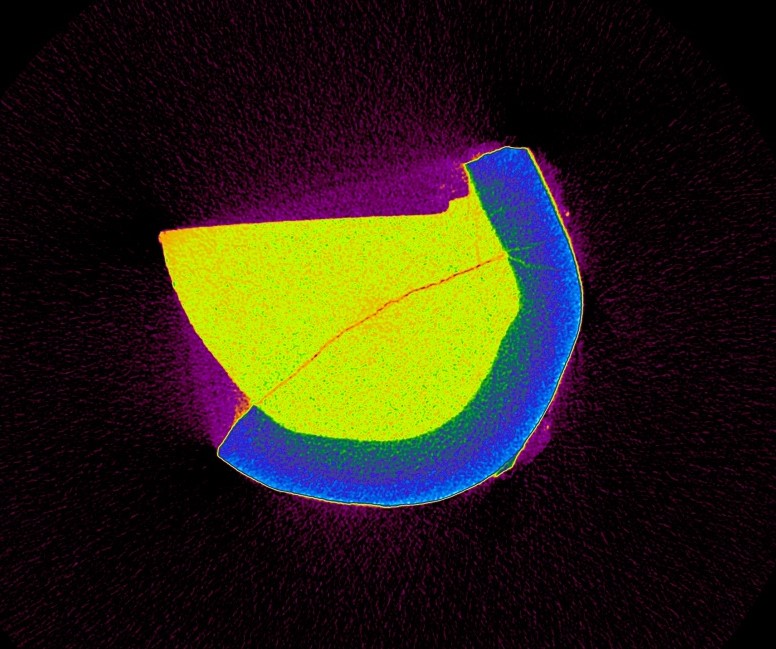


*Fig. S4:* Micro-Computed Tomography images of ICON resin infiltrants showing penetration depth (arrows showing) on teeth surface.
