# Supplementary material for: In-vitro comparative thermo-chemical aging and penetration analyses of bioactive glass-based dental resin infiltrates
Source: PeerJ. 2025 Jan 28;13:e18831. doi: 10.7717/peerj.18831 (PMC11784535; doi:10.7717/peerj.18831)
Supplement: Supplemental Information 3 [file peerj-13-18831-s003.docx]

*Table S1*: Composition of Resin infiltrant groups along with filler

| **Groups** | **Composition** | **Filler type** | **Filler Percentage** |
| --- | --- | --- | --- |
| ICON | TEGDMA-Based + Additives | None | None |
