# Supplementary material for: In-vitro comparative thermo-chemical aging and penetration analyses of bioactive glass-based dental resin infiltrates
Source: PeerJ. 2025 Jan 28;13:e18831. doi: 10.7717/peerj.18831 (PMC11784535; doi:10.7717/peerj.18831)
Supplement: Supplemental Information 4 — The last two columns show the Microleakage and Penetration depth values for ICON. [file peerj-13-18831-s004.docx]

*Table S2*: Analytic Microhardness and Surface Roughness values of All the Resin Infiltrated teeth samples with Mean and Standard Deviation after immersing into the Chemical ageing solution and Thermocycling process. The last two columns show the Microleakage and Penetration depth values for ICON.

| Groups | **Vickers Microhardness** | **Surface Roughness** | **Microleakage (µm)** | **Penetration Depth (µm)** |
| --- | --- | --- | --- | --- |
| Pre-Chemical ageing | 137.35±0.97 | 665.56± 19.22 | 4.25±0.14 | 4.81±0.19 |
| Post Chemical ageing | 111.9±4.33 | 969.71± 4.43 |  |  |
| Pre-Thermocycling | 157.43± 17.06 | 0.73±0.04 |  |  |
| Post-Thermocycling | 147.63± 16.0 | 0.96±0.004 |  |  |
|  | <0.001 | <0.001 |  |  |
