# Supplementary figures and images for: In-vitro comparative thermo-chemical aging and penetration analyses of bioactive glass-based dental resin infiltrates
Source: PeerJ. 2025 Jan 28;13:e18831. doi: 10.7717/peerj.18831 (PMC11784535; doi:10.7717/peerj.18831)

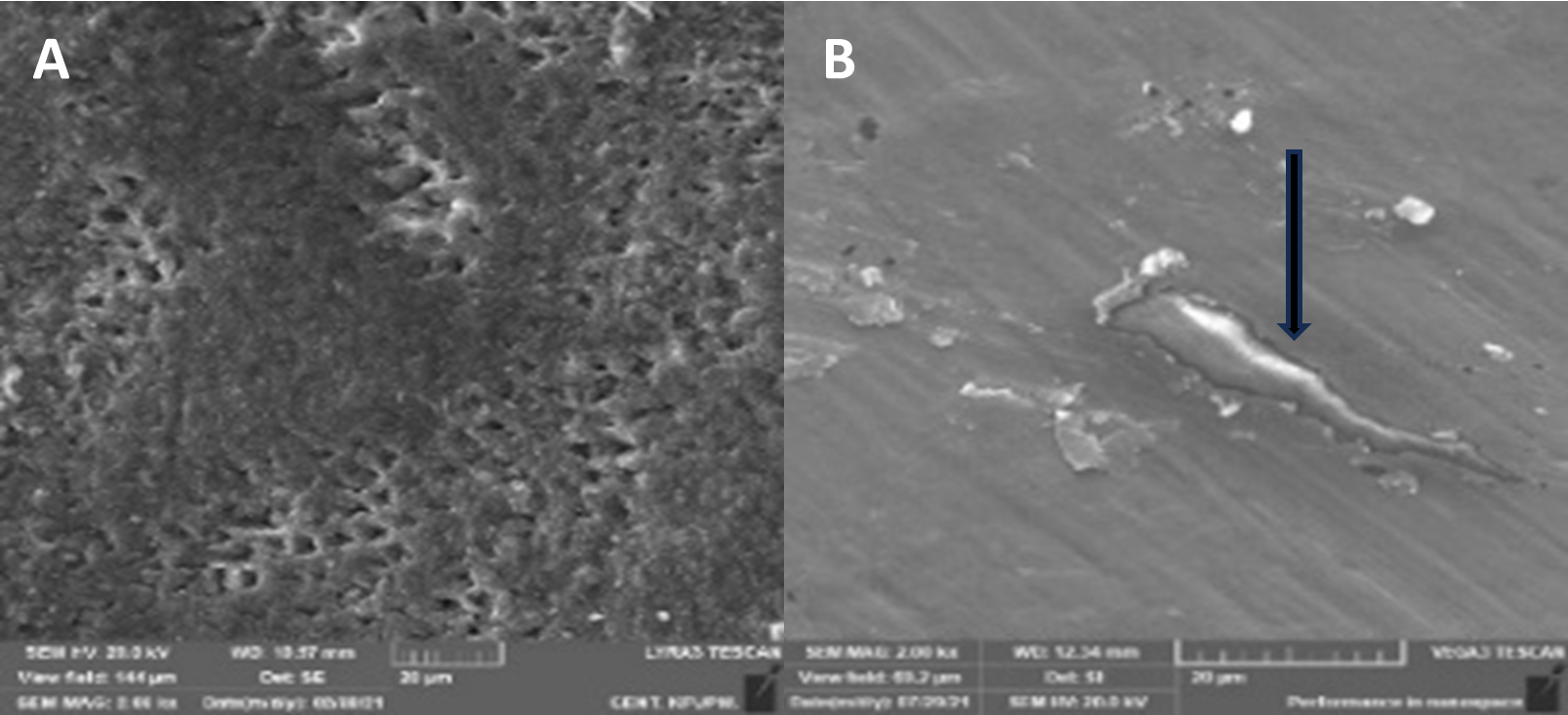

Supplement: Supplemental Information 5 — ICON resin, baseline scans before thermocycling and after thermocycling. [file peerj-13-18831-s005.png]

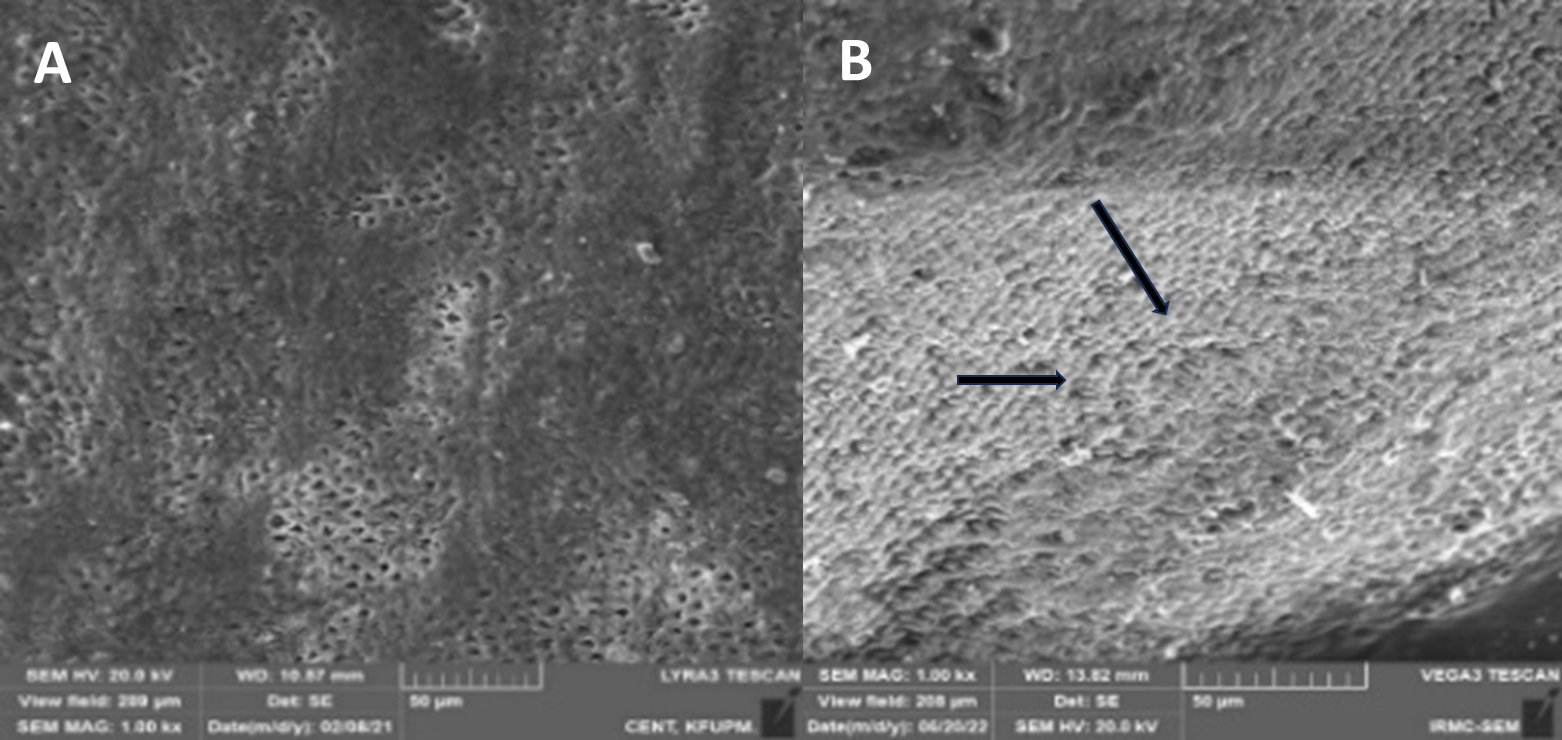

Supplement: Supplemental Information 6 — Fig. S2(A) & Fig. S2(B), ICON scans before and after immersion into the chemical solution. [file peerj-13-18831-s006.png]

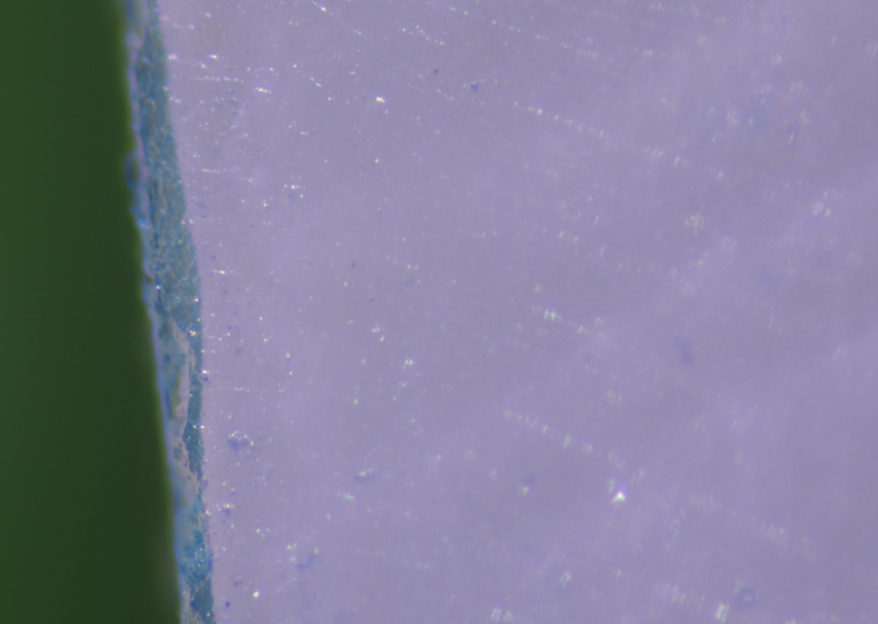

Supplement: Supplemental Information 7 — Stereomicroscope images after immersing into the Methylene Blue dye. [file peerj-13-18831-s007.png]

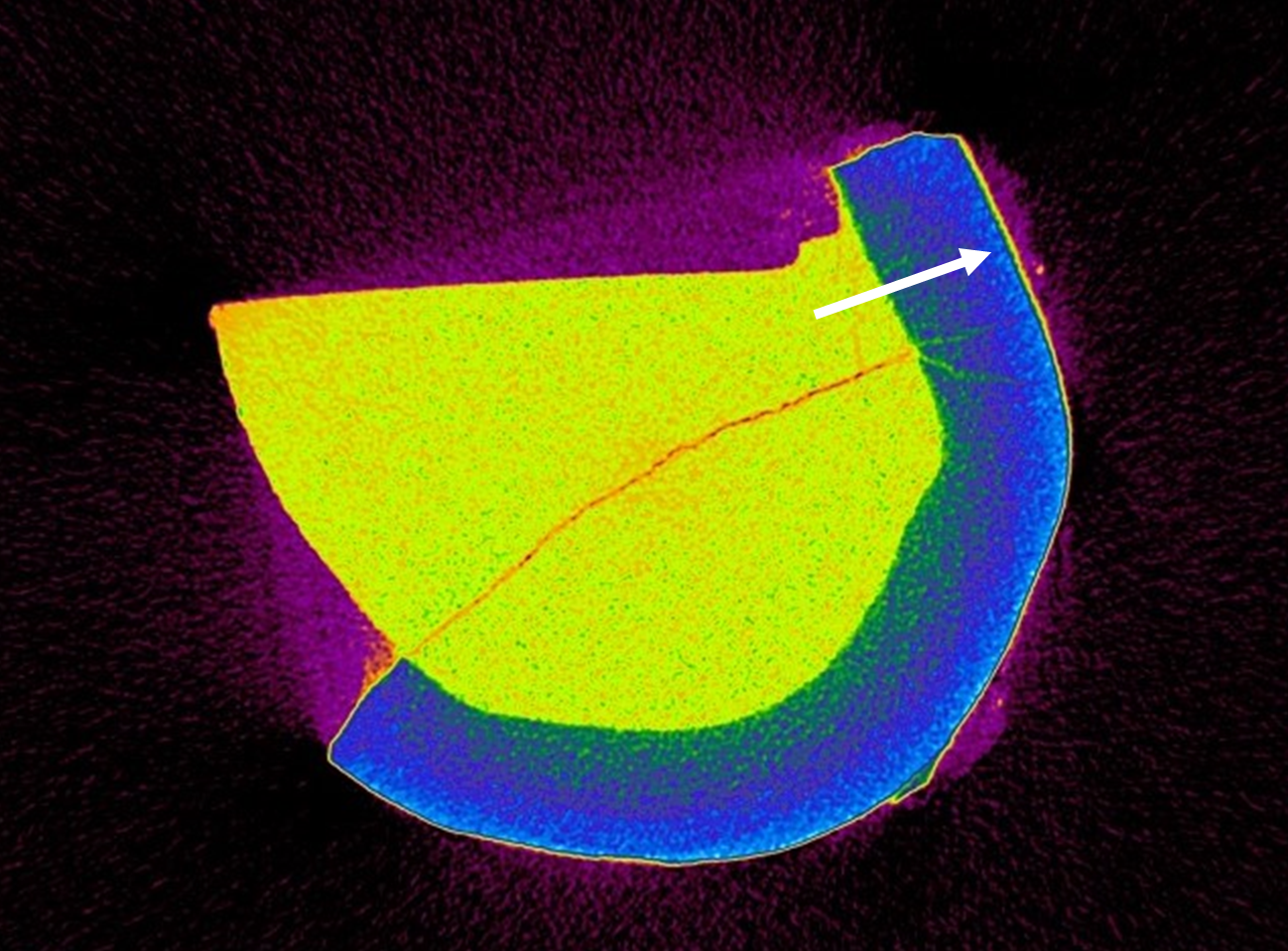

Supplement: Supplemental Information 8 — Micro-CT images of ICON resin infiltrants showing penetration depth (arrows showing) on the tooth surface. [file peerj-13-18831-s008.png]
